# Transcriptomic dataset of *Mycolicibacterium smegmatis* exposed to an imidazo[1,2-*b*][1,2,4,5]tetrazine

**DOI:** 10.1016/j.dib.2020.105805

**Published:** 2020-06-02

**Authors:** Aleksey A. Vatlin, Ksenia M. Klimina, Svetlana G. Frolova, Valery N. Danilenko, Dmitry A. Maslov

**Affiliations:** a Vavilov Institute of General Genetics Russian Academy of Sciences, Moscow, 119333, Russia; b Moscow Institute of Physics and Technology (State University), Dolgoprudny, 141701, Russia

**Keywords:** RNA sequencing, Transcriptome, imidazo[1,2-*b*][1,2,4,5]tetrazines, Mycolicibacterium smegmatis, Drug development, Tuberculosis, DEGs

## Abstract

Deciphering the mechanism of action of novel anti-tuberculosis compounds is a key step in the drug development process. We have previously described a number of imidazo[1,2-*b*][1,2,4,5]tetrazines with a promising activity on *Mycobacterium tuberculosis*[1]. These compounds had predicted activity as serine‑threonine protein kinase inhibitors, however spontaneous drug resistant *Mycolicibacterium smegmatis mc*^*2*^*155* (formerly *Mycobacterium smegmatis*) revealed only the mycobacterial mechanism of resistance to imidazo[1,2-*b*][1,2,4,5]tetrazines: mutations in *MSMEG_1380* gene lead to overexpression of the *mmpS5-mmpL5* operon in *M. smegmatis*, thus providing resistance to imidazo[1,2-*b*][1,2,4,5]tetrazines via enhanced efflux [2]. Here we report the RNA sequencing data of *M. smegmatis mc*^*2*^  *155* culture treated with one of the imidazo[1,2-*b*][1,2,4,5]tetrazines for 1.5 h and the untreated culture as a control. The mapped reads showed that a total of 1386 genes are differentially expressed in this experiment. A further analysis of these data can shed light of the mechanism of action of imidazo[1,2-*b*][1,2,4,5]tetrazines. The data generated by RNA-seq (raw reads) have been deposited to NCBI sequence read archive (SRA) and have been assigned a BioProject accession number PRJNA615922.

Specifications Table

**Table:** 

**Subject**	Biochemistry, Genetics and Molecular Biology (General)
**Specific subject area**	Transcriptomics
**Type of data**	Transcriptome sequences, tables, figure
**How data were acquired**	Illumina HiSeq 2500 sequencing platform
**Data format**	Raw Illumina HiSeq 2500 data in FASTQ format
**Parameters for data collection**	Comparison of *M. smegmatis* cultures treated with an imidazo[1,2-*b*][1,2,4,5]tetrazine with untreated control
**Description of data collection**	Total RNA extracted from six independent samples (three control replicates – untreated cultures, and three experimental replicates – treated with an imidazo[1,2-*b*][1,2,4,5]tetrazine) subjected to RNA sequencing.
**Data source location**	Vavilov Institute of General Genetics Russian Academy of Sciences, Moscow, Russia.
**Data accessibility**	Repository name: NCBI Sequence Read Archive – SRA Data identification number: PRJNA615922 Direct URL to data: https://www.ncbi.nlm.nih.gov/bioproject/PRJNA615922

Value of the Data

• These data show for the first time a transcriptomic response of *M. smegmatis* exposed to an imidazo[1,2-*b*][1,2,4,5]tetrazine - an anti-tuberculosis drug candidate.
• The data may be useful for researchers working on anti-tuberculosis drug development, as it may provide clues on imidazo[1,2-*b*][1,2,4,5]tetrazines’ mechanism of action.
• Analysis of differentially expressed genes upon exposure to imidazo[1,2-*b*][1,2,4,5]tetrazine may elucidate additional attractive biotargets in mycobacteria for drug development.

## 1 Data description

**Fig. 1:**
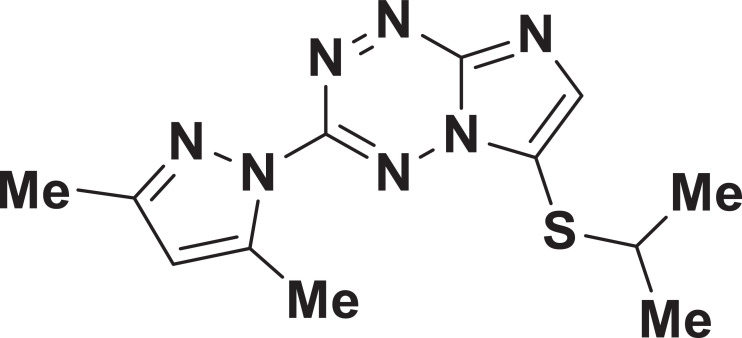
Chemical structures of the compound 3a [1].

The dataset presented in this article represents raw RNA-seq reads from samples of *Mycolicibacterium smegmatis mc^2^ 155* treated with compound **3a** (Fig. 1) – an anti-tuberculosis drug candidate of imidazo[1,2-*b*][1,2,4,5]tetrazines class [1] – at a final concentration of 256 µg/ml, and untreated control samples. The sample description together with NCBI accession numbers (BioProject, BioSample and SRA) are listed in Table 1. Sequencing and reads mapping statistics are summarized in Table 2. Reads mapping showed 1386 differentially expressed genes (DEGs) (671 downregulated and 715 upregulated) in the experimental group as compared to the control (Supplementary Table).

**Table 1:** Details of RNA-seq data submitted to the NCBI Sequence Read Archive (SRA).

Sample ID	Group	**3a** concentration	Bioproject accession no.	Biosample accession no.	SRA accession no.
1–1	Control	0 µg/ml	PRJNA615922	SAMN14476747	SRX8018106
2–1					SRX8018107
3–1					SRX8018108
4–1	Experiment	256 µg/ml		SAMN14476748	SRX8018109
5–1					SRX8018110
6–1					SRX8018111

**Table 2:** Sequencing and mapping statistics.

Sample ID	Total reads(raw)	Total reads (after trimming)	% Aligned Reads	Fragment Length (bp)
1–1	7560,080	7546,578	98,0%	219.0
2–1	7389,426	7367,050	98.2%	218.1
3–1	6420,732	6384,648	98.5%	219.4
4–1	7416,774	7396,635	98.8%	225.8
5–1	8184,700	8160,049	98.9%	222.4
6–1	8448,563	8434,466	99.1%	223.1

## 2 Experimental design, materials, and methods

### 2.1 Bacterial strains and growth conditions

*M. smegmatis mc^2^ 155* strain was used in this work. *M. smegmatis* cultures were grown in Middlebrook 7H9 medium (Difco Becton Dickinson, USA) supplemented with 0.5% (v/v) glycerol and 0.05% (v/v) Tween 80 at 37 °C and 250 rpm.

### 2.2 Experimental design

*M. smegmatis* cultures were grown overnight in Middlebrook 7H9 broth to mid-log phase (OD600 = 1.0–1.2) and then compound **3a** dissolved in DMSO was added to the medium to a final concentration of 256 μg/ml (4 × minimal inhibitory concentration [1]) for 1.5 h. The same amount of DMSO was added to the control samples. Afterwards cells were washed twice with fresh ice-cold Middlebrook 7H9 broth and total RNA was isolated. In total, 6 RNA samples were obtained — 3 biological replicates in the control and experimental conditions.

### 2.3 RNA extraction and sequencing

Cells from 10 mL culture were harvested by centrifugation for 10 min at 3000 × *g* and 4 °C, washed twice by 10 ml of fresh Middlebrook 7H9 broth and once by 1 ml of RNAprotect Bacteria Reagent (Qiagen, USA). Total RNA was extracted as described by Rustad et al. [3], with some modifications. In brief: *M. smegmatis* cells were homogenized in ExtractRNA reagent (Evrogen, Russia), followed by phenol (pH = 4.5)-chloroform/isoamyl alcohol (25:24:1) purification and precipitation with isopropanol (2:1, v/v). Remaining genomic DNA was removed by DNAse I, Amplification grade (Invitrogen, USA). Total RNA (1 µg) was used for library preparation. Ribosomal RNA was removed from the total RNA using the RiboMinus Transcriptome Isolation Kit, bacteria (Thermo Fisher Scientific) and libraries were prepared using the NEBNext^Ⓡ^ Ultra II Directional RNA Library Prep Kit (NEB), according to the manufacturer's protocol. Libraries were subsequently quantified by Quant-iT DNA Assay Kit, High Sensitivity (Thermo Fisher Scientific). Finally, equimolar quantities of all libraries (12 pM) were sequenced by a high throughput run on the Illumina HiSeq using 2 × 100 bp paired-end reads and a 5% Phix spike-in control.

### 2.4 Transcriptome data analysis

Data processing and analysis was performed as described previously by Bespyatykh et al. [4]. Raw reads’ quality was assessed by FASTQC v0.11.7 [5], the remaining adapters were removed with Trimmomatic v0.33 [6]. Reads were mapped to the *M. smegmatis mc^2^ 155* reference assembly (GCF_000015005.1) and quantified with Kallisto v0.46.0 [7]. The Degust v4.1.1 web-tool [8] with integrated edgeR v3.26.8 package [9] was used for differential expression analysis. Only genes with count per million (CPM) ≥ 1 were analyzed further. Genes were filtered based on false discovery rate cutoff (FDR) ≤ 0.05 and minimum expression fold change (FC) ≥ 2.

## Declaration of Competing Interest

The authors declare that they have no known competing financial interests or personal relationships which have, or could be perceived to have, influenced the work reported in this article.

## Appendix Supplementary materials

Image, application 1

Image, application 2
